# Supplementary material for: Identification and Validation of the Anoikis-Related Gene Signature as a Novel Prognostic Model for Cervical Squamous Cell Carcinoma, Endocervical Adenocarcinoma, and Revelation Immune Infiltration
Source: Medicina (Kaunas). 2023 Feb 13;59(2):358. doi: 10.3390/medicina59020358 (PMC9958637; doi:10.3390/medicina59020358)
Supplement: Supplementary file 1 [file medicina-59-00358-s001.zip › Figure S1.pdf]

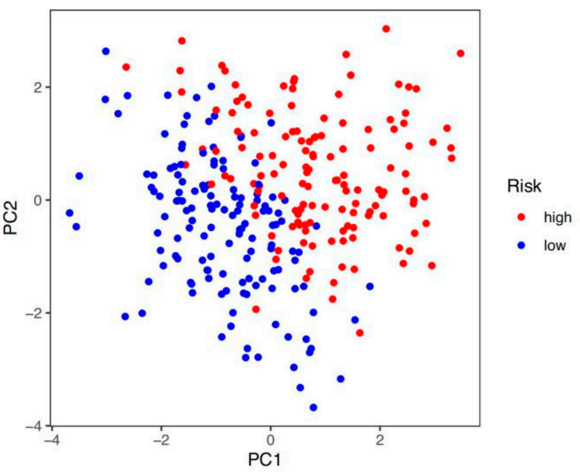

(S1)

Figure S1: The principal component analysis based on the expression of ANRGs in the different risk groups
