# Supplementary material for: Identification and Validation of the Anoikis-Related Gene Signature as a Novel Prognostic Model for Cervical Squamous Cell Carcinoma, Endocervical Adenocarcinoma, and Revelation Immune Infiltration
Source: Medicina (Kaunas). 2023 Feb 13;59(2):358. doi: 10.3390/medicina59020358 (PMC9958637; doi:10.3390/medicina59020358)
Supplement: Supplementary file 1 [file medicina-59-00358-s001.zip › Table S2.pdf]

Gene	Primer type	Primer sequence (strand)
GAPDH	Forward	5'-GGAGCGAGATCCCTCCAAAAT-3'
	Reverse	5'-GGCTGTTGTCATACTTCTCATGG-3'
ITGA5	Forward	5'-TTACGGGACTCAACTGCACC-3'
	Reverse	5'-AGCCTGAAACACTCAGCCTC-3'
ROCK1	Forward	5'-TGCCAACAGTCCTTGGGTTG-3'
	Reverse	5'-ATCGTGCCCATTTTTCAGGC-3'
HK2	Forward	5'-TTGACCAGGAGATTGACATGGG-3'
	Reverse	5'-CAACCGCATCAGGACCTCA-3'
TP53	Forward	5'-CAGCACATGACGGAGGTTGT-3'
	Reverse	5'-TCATCCAAATACTCCACACGC-3'
IKZF3	Forward	5'-ATCAACAAGGAAGGGGAGGT-3'
	Reverse	5'-GGCTCTGTGTTCTCCTCTGG-3'
ITGA5	Forward	5'-AGTTACCTTGAATTGGTTGCTGG-3'
	Reverse	5'-ATGCCATCTGTTCTCCCGTG-3'

Supplementary File 3. Table S2. Primer sequences of six ANRGs.
